# From Static to Dynamic: Complementary Roles of FSR and Piezoelectric Sensors in Wearable Gait and Pressure Monitoring

**DOI:** 10.3390/s25237377

**Published:** 2025-12-04

**Authors:** Sara Sêco, Vítor Miguel Santos, Sara Valvez, Beatriz Branquinho Gomes, Maria Augusta Neto, Ana Martins Amaro

**Affiliations:** 1 University of Coimbra, Department of Physics, 3040-516 Coimbra, Portugal; sara.seco@student.fis.uc.pt; 2 University of Coimbra, Centre of Mechanical Engineering, Materials and Processes (CEMMPRE-ARISE), Department of Mechanical Engineering, 3040-248 Coimbra, Portugal; sara.valvez@dem.uc.pt (S.V.); beatrizgomes@fcdef.uc.pt (B.B.G.); augusta.neto@dem.uc.pt (M.A.N.); ana.amaro@dem.uc.pt (A.M.A.); 3 University of Coimbra, Interdisciplinary Center for the Study of Human Performance (CIPER), Faculty of Sports Sciences and Physical Education (FCDEF), 3040-256 Coimbra, Portugal

**Keywords:** instrumented insoles, pressure sensors, prototype, compression testing, rehabilitation

## Abstract

Objective: Plantar pressure abnormalities have a significant impact on mobility and quality of life. Real-time pressure monitoring is essential in clinical and rehabilitation settings for assessing patient progress and refining treatment protocols. Instrumental and particularly smart insoles offer a promising solution by collecting biomechanical data during daily activities. However, determining the optimal combination of sensor type, number, and placement remains a key challenge for ensuring accurate and reliable measurements. This study proposes a methodology for identifying the most appropriate sensor technology for wearable insoles, with a focus on data accuracy, system efficiency, and practical applicability. Additionally, it examines the correlation between sensor signals and material behavior during compression testing. Methods: Two insole prototypes underwent compression testing: one equipped with a Force Sensitive Resistor (FSR) sensor and one with a piezoelectric sensor, both positioned at the heel. Three trials per prototype assessed consistency and repeatability. Real-time data acquisition utilized a microcontroller system, and signals were processed using a sixth-order Butterworth low-pass filter with a 5 Hz cutoff frequency to reduce noise. Results: FSR sensors demonstrated stable static responses but saturated rapidly beyond 20 N, with performance degradation observed after repeated loading cycles. Piezoelectric sensors exhibited excellent dynamic sensitivity with sharp voltage peaks but proved unable to measure sustained static pressure. Conclusions: FSR sensors are well-suited for static postural assessment and continuous pressure monitoring, while piezoelectric sensors excel in dynamic gait analysis. This comparative framework establishes a foundation for developing future smart insole systems that deliver accurate, real-time rehabilitation monitoring.

## 1. Introduction

Instrumented insoles represent a significant breakthrough in wearable health technology, offering a non-invasive and convenient method for capturing real-time biomechanical and physiological data. By integrating diverse sensor technologies into their design, these insoles facilitate ongoing analysis of plantar pressure distribution, gait dynamics, and postural control. In recent years, instrumented insoles have found widespread application in clinical settings such as diabetic foot management, postural rehabilitation, and fall prevention among the elderly, as well as in fitness and sports [1]. Their ability to continuously monitor gait symmetry, weight distribution, and balance under dynamic conditions provides valuable insights into neuromuscular control and rehabilitation progress [2]. Plantar pressure analysis has become an essential diagnostic and monitoring method for conditions like diabetic foot ulcers (DFU), musculoskeletal disorders, peripheral neuropathies, and movement disorders, including Parkinson’s and Alzheimer’s diseases. Excessive plantar pressures, particularly in the forefoot and heel, are associated with skin breakdown and ulcer development, underscoring the importance of precise, ongoing monitoring in clinical settings [2,3,4].

Pressure sensors are key to these systems because they turn mechanical forces into electrical signals that can be measured and analyzed [2]. Common types are Force-Sensitive Resistors (FSR), capacitive sensors, and piezoelectric sensors [1,3]. These sensors facilitate the estimation of key biomechanical parameters, such as Center of Pressure (CoP) and Ground Reaction Force (GRF), which are essential for evaluating balance, posture, and movement [4]. Tracking CoP helps evaluate balance and disease progression, while GRF data give detailed information about walking phases and force distribution [4,5,6]. CoP trajectories are particularly useful for assessing balance control and disease progression, while GRF data provide quantitative insights into gait phases and force distribution [4,5,6].

With advances in wearable technology, two main categories of plantar sensing systems have emerged: instrumented insoles and smart insoles [6]. While both rely on similar sensing principles, they differ in autonomy, data processing capabilities, and functionality. Instrumented insoles focus primarily on biomechanical data acquisition, such as plantar pressure or GRF, for offline analysis in clinical or research environments [7,8]. Traditionally equipped with resistive or capacitive sensors, they have evolved to integrate inertial sensors (accelerometers, gyroscopes, and magnetometers) to capture dynamic information on movement and balance [9]. In contrast, smart insoles incorporate onboard microcontrollers, wireless communication modules, and real-time feedback algorithms, allowing data to be processed and transmitted instantaneously [9,10]. This integration enables autonomous operation in both clinical and daily-use contexts. Consequently, instrumented insoles remain dominant in controlled laboratory and diagnostic applications, whereas smart insoles extend to sports performance monitoring, preventive healthcare, and assistive technologies [10]. Table 1 summarizes the key distinctions between these two technologies.

Smart insole systems frequently incorporate additional sensing components, including inertial measurement units (IMUs), temperature sensors, and humidity sensors, to enhance diagnostic capabilities [10,11,12,13]. IMUs facilitate detailed analysis of gait dynamics, fall detection, and motion segmentation. Temperature and humidity sensors are essential for diabetic foot monitoring and skin health assessment [3,10]. The integration of these multimodal sensors enables more comprehensive evaluations of gait and foot health, thereby supporting improved clinical decision-making and enhancing the reliability of real-world monitoring.

Pressure sensors are the core components in both instrumented and smart insoles and are generally categorized as resistive, piezoelectric, or capacitive types [3,7]. FSR sensors function by altering resistance in response to applied force, offering cost-effective and adaptable solutions for distributed pressure mapping [8]. Piezoelectric sensors generate electrical charges when mechanically deformed, making them particularly suitable for detecting dynamic pressure changes during gait [5]. Capacitive sensors, which detect changes in capacitance between conductive plates, provide linear responses and low energy consumption, but are susceptible to humidity and electromagnetic interference [13]. Therefore, selecting the appropriate sensor type requires careful consideration of accuracy, stability, and cost in relation to the specific application.

The design of insole-based systems is contingent upon both the selection of sensor technology and the strategic deployment of sensors. High-resolution configurations utilize extensive sensor arrays distributed across the plantar surface to generate comprehensive pressure mappings. Conversely, simplified prototypes incorporate a limited number of sensors, strategically located at biomechanically significant regions such as the hallux, metatarsal heads, and heel, to balance functionality with practicality [8,10,12].

**Figure 1:**
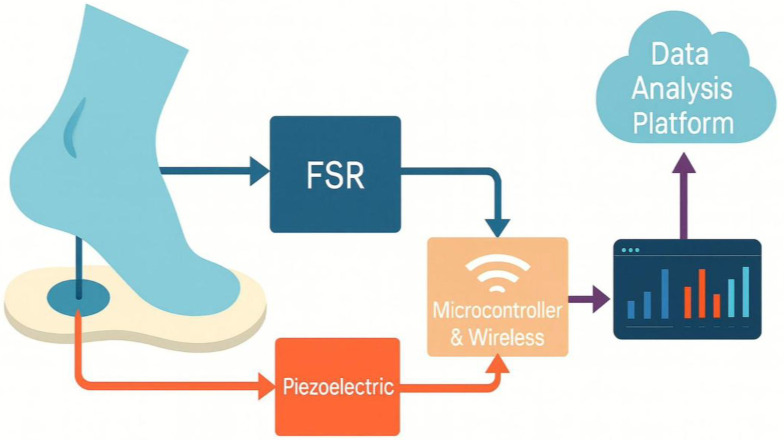
Scheme of an instrumented/smart insole functionality.

Figure 1 illustrates, in a simple and minimalistic manner, the operation of sensors attached to an insole under applied pressure. The diagram also presents the essential hardware components and software required for the equipment to function.

The scope of applications for instrumented and intelligent insoles encompasses healthcare, rehabilitation, athletic performance, and everyday activity tracking. Within the healthcare sector, these systems are essential for identifying abnormal plantar pressures, especially among diabetic patients, where ongoing monitoring can help prevent the development of ulcers and associated complications [12,14]. In rehabilitation, they allow real-time tracking of gait and posture parameters, facilitating patient progress evaluation and treatment optimization [5,10]. In sports, the ability to measure ground reaction forces, pressure distribution, and gait symmetry supports performance enhancement and injury prevention strategies [10]. The combination of portability, affordability, and sensor versatility makes smart insoles a key tool for both clinical and real-world biomechanics research.

Despite growing research efforts, few studies have experimentally validated the use of low-cost FSR and piezoelectric sensors under controlled mechanical loading conditions relevant to rehabilitation. Existing research relies heavily on computational modelling or commercial systems, often lacking standardized testing protocols and reproducible experimental validation. Addressing this gap is crucial for establishing consistent and comparable data for wearable gait monitoring systems. This study aims to experimentally compare the performance of FSR and piezoelectric sensors embedded in an insole under compressive load, evaluating their sensitivity, repeatability, and suitability for plantar pressure monitoring in clinical rehabilitation settings. The results provide a methodological foundation for developing an insole system with highly precise static and dynamic sensing capabilities, thereby enhancing the accuracy and clinical utility of wearable gait monitoring technologies.

## 2. Experimental Procedure

### 2.1. Materials

**Figure 2:**
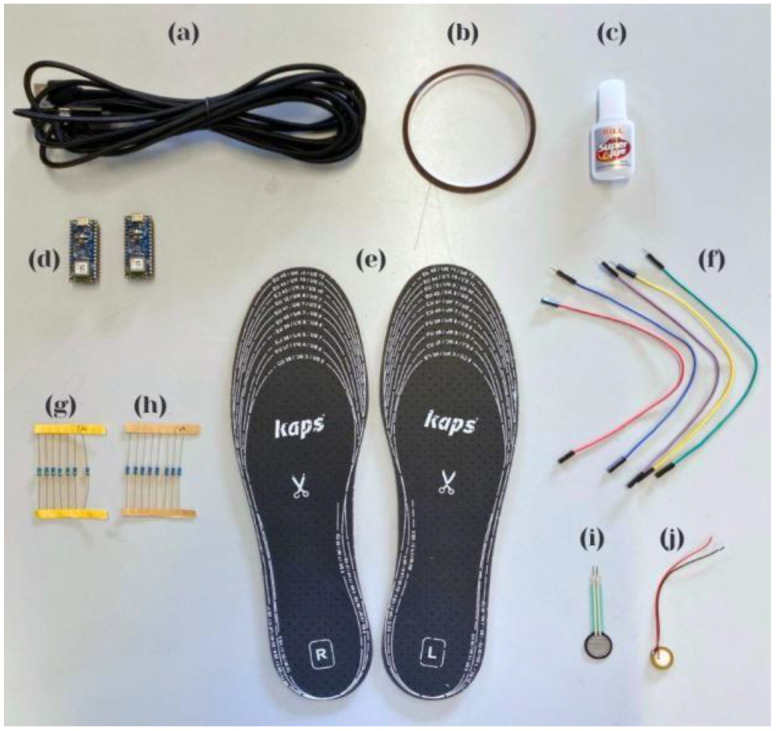
Components used in the construction of the prototype and the data acquisition system, including a USB cable (**a**), Kapton tape (**b**), Instant glue (**c**), two microcontrollers (**d**), pair of size 46 insoles, left and right (**e**), jumper wires (**f**), 1 MΩ resistors (**g**), 10 kΩ resistors (**h**), an FSR402 sensor (**i**), and a piezoelectric sensor (**j**).

The materials utilized in the construction of the prototype are depicted in Figure 2 and are further elucidated in this section. The figure delineates the essential components of the instrumented insole, including the foundational substrate, sensor layers, wiring configuration, and encapsulation materials. Each component was chosen to optimize flexibility, durability, and the precise transmission of load to the sensing elements under compressive forces. The accompanying description provides a comprehensive overview of the composition and functional role of each material, thereby facilitating the attainment of the desired mechanical and operational performance of the prototype.

The components include a USB cable (Figure 2a) for power and data transfer; Kapton tape (Figure 2b) for temporary fixation and electrical insulation; instant glue (Figure 2c) for permanent bonding of sensor elements; two microcontrollers (Figure 2d); a pair of size 46 insoles, left and right (Figure 2e); jumper wires (Figure 2f) for electrical connections; 1 MΩ resistors (Figure 2g) and 10 kΩ resistors (Figure 2h) for signal conditioning; an FSR402 sensor (Figure 2i); and a piezoelectric sensor (Figure 2j). These components were selected to ensure proper integration, signal reliability, and mechanical compatibility with flexible wearable applications.

### 2.2. Methods

#### 2.2.1. Sensor Selection and Configuration

FSR and piezoelectric sensors were selected for this study due to their complementary characteristics and widespread application in plantar pressure monitoring systems. These sensor types are frequently cited in the literature for their practicality, accessibility, and effectiveness in wearable insole applications [5]. FSR sensors offer advantages such as low cost, thin and flexible construction, and user comfort, making them well-suited for integration into insoles without disrupting natural gait patterns [7].

Piezoelectric sensors exhibit superior responsiveness to transient forces, a broad measurement range, and high sensitivity, which are advantageous for detecting impact events and dynamic gait phases [10]. Although they are less effective for static pressure detection, their excellent dynamic performance supports their inclusion in applications requiring temporal precision [9]. By studying the strengths of FSR and piezoelectric technologies, this work adopts a sensor configuration consistent with current research trends, ensuring a balanced and robust approach to plantar pressure assessment.

To achieve the study main objective while maintaining a simple and efficient design, only one sensor was integrated into each insole. This choice reduced system complexity, energy consumption, and cost while still capturing the most relevant data for compression testing [10].

Given the single-sensor configuration, selecting the optimal placement was essential. The heel was chosen because it typically bears the highest pressure during both standing and walking. This region is commonly prioritized in simplified pressure measurement systems, providing reliable detection of key force patterns without requiring multiple sensors [15].

#### 2.2.2. Prototype Construction

**Figure 3:**
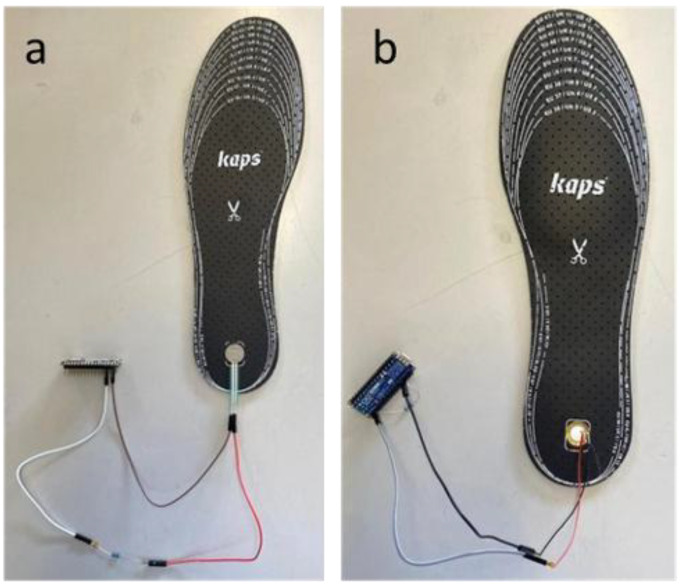
Insoles Prototype: (**a**) FSR Sensor Prototype and (**b**) Piezoelectric Sensor Prototype.

Two prototypes were developed. The first prototype (Figure 3a) involved attaching a FSR sensor (Figure 2i) to the heel area of the left insole (Figure 2e) using instant glue (Figure 2c). The second prototype (Figure 3b) followed the same process, using a piezoelectric sensor (Figure 2j) attached to the heel area of the right insole (Figure 2e). After fixing the sensors, both prototypes were connected to a microcontroller (Figure 2d).

In the FSR prototype (left insole, Figure 3a), the connection to the microcontroller was established using one female-to-female and two male-to-female jumper wires (Figure 2f). A 10 kΩ resistor (Figure 2h) was incorporated into the circuit to form a voltage divider, enabling analog input readings of the FSR variable resistance. The connections were temporarily secured with Kapton tape (Figure 2b). In the piezoelectric prototype (right insole, Figure 3b), the sensor was connected using two male-to-female jumper wires (Figure 2f), which are compatible with the microcontroller interface. A 1 MΩ resistor (Figure 2g) was added in series to limit current and protect the microcontroller from potential voltage spikes caused by the sensor’s rapid charge and discharge behaviour.

In both prototypes, jumper wires were directly connected to designated microcontroller pins via small interface boards. The microcontroller was powered and communicated with through a USB-to-micro-USB cable (Figure 2a) connected to the computer, with the corresponding port configured in the Arduino IDE software (version 2.3.6).

### 2.3. Prototype Validation

To validate the functionality and responsiveness of the prototype, a compression test with in-situ data collection was performed. This method enabled the simultaneous application of mechanical loads and real-time monitoring of the electrical signals generated by the embedded FSR and piezoelectric sensors. By simulating localized pressures similar to those experienced during walking or standing, the tests offered valuable insights into sensor performance, signal stability, and mechanical integration within the insole. The data collected confirmed the reliability of the prototypes for future use in gait analysis and rehabilitation monitoring.

With the data acquisition system properly initialized and the sensors securely integrated into the insoles, compression tests were performed to evaluate the performance of both prototypes under mechanical pressure. These tests are crucial for understanding how each sensor reacts to real-world conditions, including the forces typically experienced during walking or standing. Before testing, both the FSR and the piezoelectric sensor underwent individual calibration procedures to ensure accurate and reliable measurements. This process involved establishing a zero-load baseline followed by applying calibrated weights of known mass to the sensors. The electrical outputs corresponding to each applied load were recorded, helping to create a calibration curve that links sensor signals with the forces applied. This calibration allows for precise interpretation of sensor data during future compression tests.

#### 2.3.1. Mechanical Characterization

The compression test was conducted using a Shimadzu Autograph AG-X equipped with a 5 kN load cell, operating at a strain rate of 1 mm/min at room temperature. Two samples were tested under identical conditions, with each sample undergoing three repetitions separated by short intervals. These repetitions helped evaluate the consistency of the sensor’s responses, ensuring reliability and reducing the influence of random fluctuations, thus providing a clearer picture of the sensor’s performance. During each test, controlled deformation was applied directly to the heel area of the insole where the sensor was embedded. The sensor’s electrical signals were monitored through a microcontroller connected via a USB cable to a computer (Apple, CA, USA, MacBook Pro 13-inch, M2, 2022; macOS Sequoia 15.4.1).

#### 2.3.2. Data Acquisition and Processing

Before the compression experiments, a data acquisition and processing system was established to facilitate real-time monitoring of the electrical signals produced by the integrated FSR and piezoelectric sensors. The microcontroller employed in this study was connected to a computer via a USB cable, thus providing both power and data communication. This specific microcontroller model was selected due to its intuitive and user-friendly software environment. Among platforms compatible with TinyML applications, Arduino is one of the most widely utilized owing to its accessibility, ease of use, and robust community support. The microcontroller was connected to the external sensors (FSR and piezoelectric) via jumper wires. To ensure compatibility with the chosen microcontroller, the appropriate board package software was installed, enabling existing code to accurately read the sensor signals. This code was sourced from the open-source platform GitHub (version 3.5.4) to enable real-time data acquisition. It was subsequently uploaded to the microcontroller via the Arduino IDE and executed during testing procedures. A custom Python (version 3.13.4) script was developed to capture the serial output from the microcontroller and automatically save it as a Comma-Separated Values (CSV) file upon each execution. These CSV files were subsequently imported into MATLAB (R2025a) for visualization and analysis. The resulting plots were utilized to evaluate the dynamic behavior and performance of both sensors under mechanical loading.

Three compression tests were conducted for each prototype, consisting of an insole integrated with either an FSR or a piezoelectric sensor: the experimental protocol aimed to replicate physiologically relevant loading conditions while maintaining rigorous control over testing parameters.

The raw voltage signals acquired during testing were compromised by high-frequency noise, attributed primarily to electrical interference and the use of Kapton tape for component connections rather than soldered joints. This assembly approach, while enabling rapid prototyping, introduced contact resistance variability that manifested as signal instability. To address this issue, a sixth-order low-pass Butterworth filter was applied using zero-phase digital filtering (MATLAB’s filtfilt function) with a cutoff frequency of 5 Hz, adjusted to each trial’s sampling rate.

The Butterworth filter was selected for its maximally flat passband response, which preserves signal amplitude characteristics while effectively attenuating high-frequency noise components. This filter design is well-established in biomedical signal processing, demonstrating efficacy in applications ranging from respiration monitoring to gait analysis [16,17]. The zero-phase filtering approach ensures that temporal relationships within the signal are preserved, which is a critical consideration when correlating sensor responses with mechanical loading events.

The following sections present a comprehensive analysis of sensor electrical responses and their correlation with mechanical compression behavior. This integrated approach enables the identification of the operational characteristics, performance limitations, and potential clinical applications of each sensor type.

## 3. Results and Discussion

### 3.1. Mechanical Response Recorded by Sensors

#### 3.1.1. FSR Sensor Response

**Figure 4:**
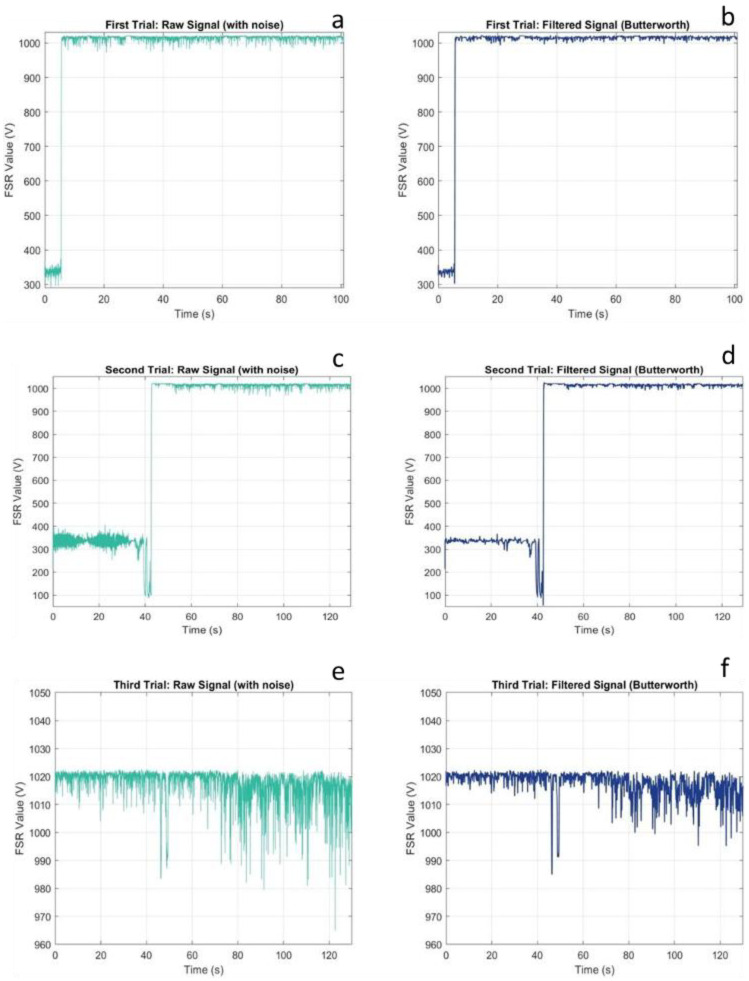
FSR sensor voltage responses showing raw (**a**,**c**,**e**) and filtered (**b**,**d**,**f**) signals for three compression trials.

Figure 4 presents the voltage response characteristics of the FSR sensor across three successive compression trials, displaying both raw and filtered signals to illustrate the effectiveness of noise reduction preprocessing.

The FSR sensor exhibited a non-zero baseline voltage even under no-load conditions, a characteristic that requires careful consideration in calibration protocols. The offset voltage, ranging from approximately 0.05 to 0.15 V across trials, is attributed to the temporary wire connections secured with Kapton tape. Unlike soldered connections, which provide stable electrical contact, tape-based connections introduce variable contact resistance that fluctuates with minor mechanical disturbances. This phenomenon has significant implications for sensor deployment: while the offset itself can be compensated through baseline subtraction, the underlying contact instability may contribute to measurement uncertainty during dynamic loading.

Despite this baseline artifact, the sensor demonstrated reliable transition detection between unloaded and loaded states. Upon load application, the FSR exhibited characteristic rapid voltage increase, reaching saturation within 2–3 s. This saturation behaviour is inherent to the sensor’s design specifications, which define a nominal operating range of 0.2 to 20 N. The applied compressive load clearly exceeded this threshold, driving the sensor into its saturation regime where further load increases produce negligible voltage changes.

From a clinical monitoring perspective, this saturation characteristic presents both limitations and opportunities. The limited dynamic range constrains the sensor’s utility for quantifying high-magnitude plantar pressures, such as those encountered during running or in individuals with elevated body mass [18,19]. However, the stable saturated output provides a reliable binary indicator of sustained load presence, a feature potentially valuable for applications such as standing posture monitoring or detection of prolonged pressure exposure in diabetic foot care, where the primary concern is identifying areas experiencing continuous loading rather than precise force quantification [20,21]. The second compression trial revealed progressive performance degradation, characterized by increased signal noise and a prolonged onset time of saturation. This comportment is characteristic of polymer-based resistive sensors subjected to repeated mechanical stress. The conductive polymer composite that forms the sensing element experiences microstructural changes under cyclic loading: polymer chains may undergo plastic deformation, conductive particle networks can be disrupted, and adhesive layer integrity may degrade [22]. These mechanisms collectively reduce sensor responsiveness and increase susceptibility to noise [23]. The extended saturation onset observed in the second trial-approximately 5 s compared to 2–3 s in the first trial, suggests diminished sensitivity to applied force. This nonlinear degradation pattern indicates that the sensor’s transduction mechanism was compromised, potentially through partial delamination of sensing layers or alteration of the conductive particle network. The absence of soldered connections likely exacerbated this degradation by allowing micromotion at electrical interfaces, introducing additional mechanical stress cycles beyond the intended compressive loading [23,24]. By the third trial, the sensor exhibited complete saturation throughout the measurement period, indicating irreversible damage to the sensing element. This failure mode, characterized by sustained maximum output regardless of applied load, renders the sensor incapable of distinguishing between different loading states. Such performance represents complete loss of measurement functionality and would necessitate sensor replacement in any practical application.

These findings underscore critical considerations for FSR sensor deployment in rehabilitation monitoring systems. First, sensor durability under repeated loading must be carefully evaluated relative to intended application duration and loading frequency. Short-term monitoring applications may tolerate gradual sensitivity degradation, while long-term continuous monitoring would require periodic sensor replacement or more robust sensor technologies. Second, signal conditioning and connection methods significantly impact measurement reliability. Future implementations should prioritize soldered connections or alternative secure fastening methods to minimize contact resistance variability. Third, integration of accelerometers or other motion sensors could enable detection of wire movement or mechanical disturbances, allowing differentiation between true pressure signals and motion artifacts [25,26].

#### 3.1.2. Piezoelectric Sensor Response

**Figure 5:**
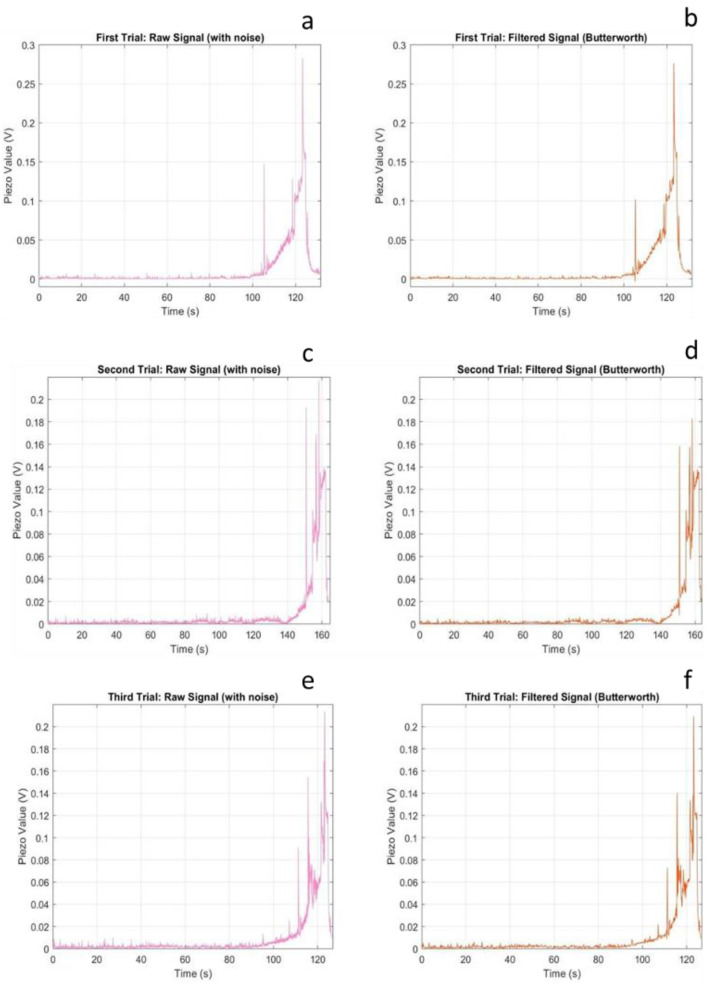
Piezoelectric sensor voltage responses showing raw (**a**,**c**,**e**) and filtered (**b**,**d**,**f**) signals for three compression trials.

Figure 5 presents the electrical response characteristics of the piezoelectric sensor across three compression trials, revealing markedly different behavior compared to the FSR sensor.

First, it is important to clarify that to ensure consistent comparison between material deformation and sensor output, both trials were analysed over the same time interval, given the longer response duration in the second test.

Regarding the data gathered in the trials, the piezoelectric sensor exhibited fundamentally different operational characteristics compared to the FSR, originating from distinct transduction mechanisms. While FSR sensors measure quasi-static force through resistance changes, piezoelectric sensors respond to dynamic mechanical stress by generating transient electrical charges. This distinction is evident in the observed signal patterns: the piezoelectric sensor produces sharp, well-defined voltage peaks that coincide with rapid load changes, while maintaining a near-zero baseline output during steady-state conditions [27,28].

The sensor’s initial response in the first trial demonstrated its optimal operating characteristics. Starting from a true zero baseline with minimal noise oscillations, the sensor generated distinct voltage spikes reaching peak amplitudes of approximately 1.5–2.0 V upon load application. These peaks exhibited rapid rise times (<0.5 s) and equally rapid decay, reflecting the sensor’s inherent high-pass filtering behavior. The piezoelectric effect generates charge proportional to the rate of mechanical strain rather than absolute strain magnitude, explaining why the sensor responds primarily to transient loading events rather than sustained pressure [9].

The second trial revealed important temporal dynamics in the performance of piezoelectric sensors. The voltage peaks appeared with greater temporal separation and demonstrated more gradual rise characteristics compared to the first trial. This alteration can be attributed to the extended rest interval between trials, which allowed complete dissipation of residual charge within the piezoelectric element and capacitance in the measurement circuit. When the sensor begins from a fully discharged state, the initial response to loading exhibits modified characteristics as the charge distribution re-establishes within the piezoelectric material. The third trial demonstrated remarkable consistency with previous measurements, with only minor variations in peak timing and amplitude. This reproducibility stands in stark contrast to the progressive degradation observed in the FSR sensor, reflecting the fundamental robustness of the piezoelectric transduction mechanism. Piezoelectric materials generate an electrical response through deformation of their crystalline structure, rather than relying on polymer chain mobility or conductive particle networks. This mechanism exhibits minimal hysteresis and fatigue under moderate loading conditions, contributing to excellent long-term stability [29,30].

The observed signal consistency across trials provides strong evidence for the suitability of piezoelectric sensors in applications requiring repeated measurements over extended periods. While the FSR demonstrated significant performance degradation after just three loading cycles, the piezoelectric sensor maintained its response characteristics, suggesting potential for thousands of loading cycles before considerable performance degradation occurs. This durability advantage is particularly relevant for continuous gait monitoring applications where sensors may experience hundreds to thousands of loading cycles daily. However, the piezoelectric sensor’s fundamental limitation, inability to measure static loads, constrains its application scope. The sensor’s high-pass filtering characteristic means that a constant pressure, regardless of its magnitude, produces a zero-output voltage. This behavior makes piezoelectric sensors unsuitable for applications requiring sustained pressure monitoring, such as evaluating standing posture or detecting prolonged localized pressure in diabetic foot care. The sensor’s optimal application domain is in detecting transient events, such as heel strikes, toe-offs, sudden weight shifts, and other dynamic loading transitions that characterize gait and movement analysis.

### 3.2. Prototypes Compression Response

The analysis of stress-strain relationships for both prototypes provides insight into the mechanical behavior of the insole substrate and its interaction with embedded sensors. These measurements establish baseline mechanical properties essential for interpreting sensor responses and evaluating prototype suitability for plantar pressure monitoring applications.

**Figure 6:**
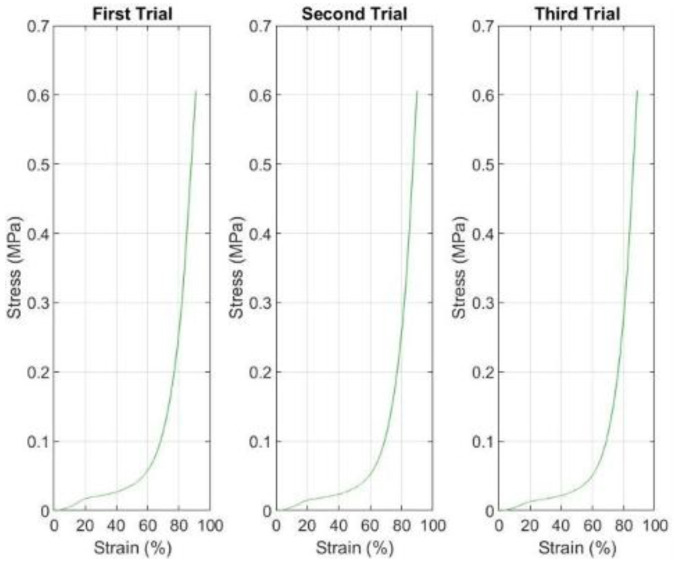
Stress-strain curves for FSR prototype showing consistent mechanical response across three trials.

Figure 6 presents the stress-strain curves for the FSR-equipped prototype across three compression trials. The curves exhibit characteristic behavior of closed-cell foam materials commonly used in footwear applications: an initial low-modulus region where cell walls bend and buckle, followed by a transition to higher stiffness as cells collapse and material densification occurs.

The three trials demonstrated remarkable consistency, with curves nearly superimposing throughout the loading range. This reproducibility indicates that the insole material experienced minimal permanent deformation, with elastic recovery occurring effectively between trials. The absence of significant hysteresis or residual strain suggests that the material remained within its elastic operating regime, an essential characteristic for sensors intended for repeated use.

A minor discontinuity observed around 20 s in all three trials corresponds to a momentary clamp adjustment during testing. This artifact, while producing a small perturbation in the stress-strain relationship, does not compromise overall data integrity. More significantly, the calculated stress values should be interpreted with appropriate consideration of measurement limitations. The nominal contact area used for stress calculation was based on the clamp geometry rather than the actual contact area between clamp and insole surface. Given the compressible nature of the insole material, the true contact area is likely increased during compression, suggesting that reported stress values may overestimate actual stress magnitudes. However, since this systematic error affects all measurements consistently, relative comparisons between trials and between sensor types remain valid.

**Figure 7:**
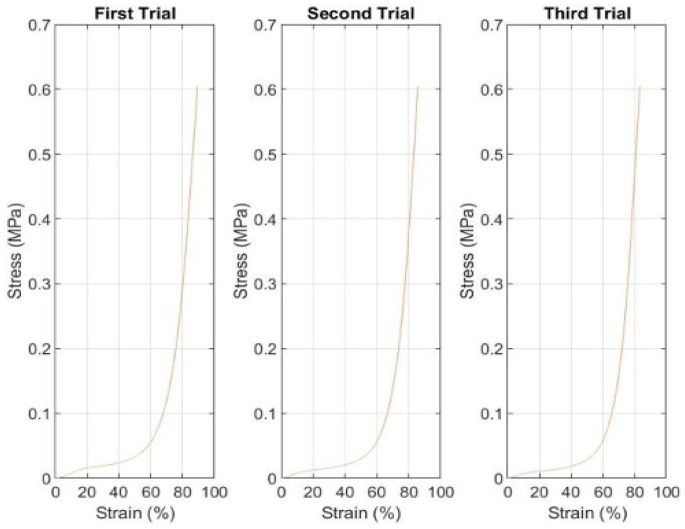
Stress-strain curves for piezoelectric prototype showing mechanical response characteristics.

Figure 7 presents corresponding stress-strain data for the piezoelectric sensor prototype. The mechanical response mirrors that of the FSR prototype, exhibiting the same characteristic two-phase behavior: initial compliant response followed by stiffening at higher strains.

Interestingly, the piezoelectric prototype exhibited slightly greater temporal spacing between consecutive compression cycles compared to the FSR prototype. This observation may reflect subtle differences in insole material properties resulting from manufacturing variability. Even nominally identical insoles produced through commercial manufacturing processes can exhibit variations in foam density, cell structure, or thickness distributions. Such variations, while typically small, can influence mechanical response characteristics, particularly the rate of elastic recovery following compression.

Alternative explanations for the temporal variation include differences in experimental protocol execution, such as variations in the rest period between trials or minor differences in clamp positioning. The operator’s manual control of timing and positioning introduces inherent variability that, while minimized through standardized procedures, cannot be eliminated without fully automated testing protocols.

Despite these temporal variations, the three trials for the piezoelectric prototype demonstrated excellent consistency in mechanical response magnitude and curve shape. This consistency reinforces the conclusion that both insole materials exhibited stable mechanical behavior throughout testing, providing reliable platforms for sensor evaluation. The insoles’ ability to recover elastically between loading cycles without significant property changes suggests they would maintain functional performance through multiple use cycles in practical applications.

### 3.3. Integrated Sensor-Substrate Response Analysis

The relationship between sensor electrical outputs and mechanical loading of the insole substrate provides critical insight into each sensor type’s operational characteristics and application suitability. By correlating these signals, we can identify how effectively each sensor transduces mechanical stimuli into electrical signals and understand the implications for rehabilitation monitoring applications.

#### 3.3.1. FSR Sensor-Substrate Correlation

**Figure 8:**
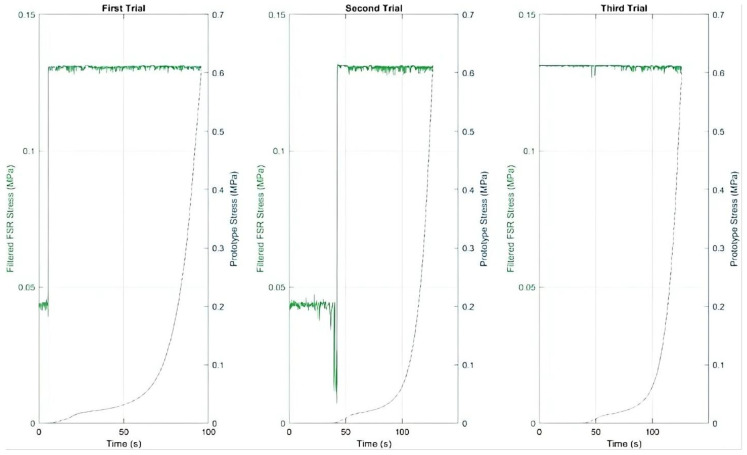
FSR sensor voltage (**left axis**) and mechanical stress (**right axis**) showing saturation behavior across three trials.

Figure 8 presents the simultaneous measurement of FSR voltage output (left axis) and applied mechanical stress (right axis) throughout three compression trials. This dual-axis representation enables direct visualization of the sensor’s response characteristics relative to physical stimulus.

The initial portion of each curve demonstrates a consistent increase, indicating that the sensor responds coherently to the onset of material deformation. In each trial, however, the FSR signal reaches its saturation limit relatively quickly, while the mechanical stress continues to increase. This saturation is consistent across trials and reflects the known limitation of FSR, which provide reliable measurements only within a limited load range (approximately 0.2 to 20 N). Beyond this range, the sensor output stabilizes and does not reflect further increases in mechanical stress.

Despite this limitation in measurement range, the sensor maintains a stable output level once saturated, which can still serve as a useful indicator of sustained load presence.

#### 3.3.2. Piezoelectric Sensor-Substrate Correlation

**Figure 9:**
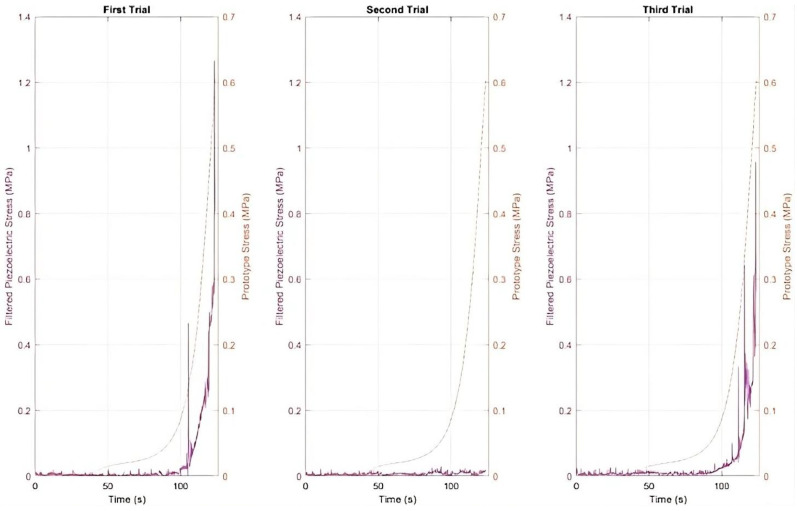
Piezoelectric sensor voltage spikes (**left axis**) corresponding to stress application events (**right axis**).

Figure 9 presents the filtered voltage signal from the piezoelectric sensor (left *Y*-axis) alongside the mechanical stress applied to the insole (right *Y*-axis), both measured in MPa and plotted over time for three separate trials.

Mechanical stress increases progressively and steadily throughout each trial, while the piezoelectric signal exhibits sharp, well-defined peaks that appear abruptly and briefly. This pattern reflects the sensor’s sensitivity to sudden changes in load rather than to sustained force.

In the first trial, the peaks occur precisely when the mechanical stress reaches its maximum values, highlighting the responsiveness of the piezoelectric sensor to transient load variations. In the second trial, these peaks appear later despite similar stress levels. This delay may be attributed to minor variations in the interface between the piezoelectric sensor and the insole, such as differences in alignment, contact pressure distribution, or positioning, or to the rest period between trials affecting the sensor’s internal charge accumulation.

Across all trials, the piezoelectric sensor does not produce a continuous or saturated output like the FSR sensor but instead responds discreetly to dynamic load changes. This behavior confirms the piezoelectric sensor’s effectiveness for detecting impacts or sudden load fluctuations and indicates its unsuitability for measuring sustained loads or static deformation.

## 4. Conclusions

This study compared the performance of FSR and piezoelectric sensors integrated into commercially available insoles through controlled compression testing. This experiment also serves as a preliminary proof of concept and highlights the necessity for higher-range sensing elements to ensure reliable operation in real in-shoe scenarios under human body load.

FSR sensors demonstrated reliable static pressure detection and maintained stable output under sustained loading conditions, making them well-suited for postural stability assessment, standing balance evaluation, and diabetic foot pressure monitoring. However, they exhibited limited dynamic range with saturation occurring at approximately 20 N and showed progressive performance degradation under repeated loading cycles. These limitations necessitate careful consideration of expected loading magnitudes and establishment of sensor replacement intervals for long-term monitoring applications.

Piezoelectric sensors exhibited exceptional dynamic sensitivity, generating sharp, reproducible voltage peaks synchronized with transient loading events. Their high temporal resolution, robust repeatability across multiple loading cycles, and minimal performance degradation establish their suitability for dynamic gait analysis, temporal event detection, and activity classification. However, their fundamental inability to measure static loads constrains their application to dynamic monitoring scenarios where temporal event detection takes priority over sustained pressure quantification.

The complementary nature of these sensor technologies motivates development of hybrid system architectures that leverage both capabilities within a single platform. Such systems could employ piezoelectric sensors at high-impact regions for precise temporal event detection while positioning FSR sensors at other locations for sustained pressure monitoring. This integrated approach would enable extraction of comprehensive biomechanical parameters including both temporal gait metrics and spatial pressure distributions.

The experimental methodology employed in this study provides a reproducible framework for sensor validation that addresses gaps in existing literature, where characterization often relies on computational modelling or proprietary systems. Several practical considerations emerged that inform future development: production systems require soldered connections rather than temporary fastening methods to ensure signal integrity, durability assessment protocols must simulate realistic use conditions to establish maintenance intervals, and signal conditioning strategies should be implemented to enhance measurement accuracy in uncontrolled environments.

The next development phase involves implementing multi-sensor arrays with four sensors per insole positioned at biomechanically relevant locations to capture comprehensive plantar pressure distributions. These enhanced prototypes will undergo validation testing including both controlled mechanical compression and human subject trials during actual gait and standing activities to confirm that laboratory-characterized performances translate to real-world applications.

In conclusion, FSR and piezoelectric sensors exhibit complementary characteristics that position them for synergistic roles in rehabilitation monitoring. Rather than competing alternatives, optimal system design embraces both technologies within hybrid architectures that capture comprehensive biomechanical information. The methodological framework and technical insights presented here provide a foundation for developing next-generation smart insole systems that enhance clinical decision-making, enable personalized rehabilitation interventions, and ultimately improve patient outcomes by bridging the gap between laboratory research and clinical practice.

## Figures and Tables

**Table 1:** Features Comparison between Instrumented Insoles and Smart Insoles.

	Instrumented Insoles	Smart Insoles
**Purpose**	Objective data acquisition (e.g., pressure, temperature, motion) for later analysis.	Real-time data acquisition, processing, and instantaneous feedback or key insights.
**Sensors**	Pressure, temperature, humidity, IMU (accelerometer, gyroscope, magnetometer).	Same sensors are integrated with onboard processing for real-time motion analysis and adaptative feedback.
**Data Processing**	External (e.g., PC).	Embedded processing takes place either inside the equipment or through a paired mobile device.
**Applications**	Clinical diagnostics, gait analysis, and rehabilitation.	Preventive care, sports performance, fall detection, and adaptative real-time feedback.
**Battery Duration**	Limited (often requires a wired connection or external devices).	High (wireless and continuous operation via Bluetooth or Wi-Fi).

## Data Availability

All the gathered and analyzed data is shared in the present work.
